# Addressing systemic problems with exposure assessments to protect the public’s health

**DOI:** 10.1186/s12940-022-00917-0

**Published:** 2023-01-12

**Authors:** Laura N. Vandenberg, Swati D. G. Rayasam, Daniel A. Axelrad, Deborah H. Bennett, Phil Brown, Courtney C. Carignan, Nicholas Chartres, Miriam L. Diamond, Rashmi Joglekar, Bhavna Shamasunder, Kristin Shrader-Frechette, Wilma A. Subra, Ken Zarker, Tracey J. Woodruff

**Affiliations:** 1 Department of Environmental Health Sciences, School of Public Health & Health Sciences, University of Massachusetts Amherst, Amherst, MA USA; 2 Program on Reproductive Health and the Environment, Department of Obstetrics, Gynecology and Reproductive Sciences, University of California, San Francisco, CA USA; 3 Independent Consultant, Washington, DC USA; 4 Department of Public Health Sciences, University of California, Davis, Davis, CA USA; 5 Social Science Environmental Health Research Institute, Northeastern University, Boston, MA USA; 6 Department of Food Science and Human Nutrition, Department of Pharmacology and Toxicology, Michigan State University, East Lansing, MI USA; 7 Department of Earth Sciences, University of Toronto, Toronto, ON Canada; 8 School of the Environment, University of Toronto, Toronto, ON Canada; 9 Earthjustice, New York, NY USA; 10 Earthjustice, Washington, DC USA; 11 Department of Urban & Environmental Policy and Public Health, Occidental College, Los Angeles, CA USA; 12 Department of Biological Sciences, University of Notre Dame, Notre Dame, IN USA; 13 Department of Philosophy, University of Notre Dame, Notre Dame, IN USA; 14 Louisiana Environmental Action Network, Baton Rouge, LA USA; 15 Washington State Department of Ecology, Olympia, WA USA

**Keywords:** Biomonitoring, Uncertainty, Superfund, Toxic substances control act, Physiologically based toxicokinetic model, US Environmental Protection Agency

## Abstract

**Background:**

Understanding, characterizing, and quantifying human exposures to environmental chemicals is critical to protect public health. Exposure assessments are key to determining risks to the general population and for specific subpopulations given that exposures differ between groups. Exposure data are also important for understanding where interventions, including public policies, should be targeted and the extent to which interventions have been successful. In this review, we aim to show how inadequacies in exposure assessments conducted by polluting industries or regulatory agencies have led to downplaying or disregarding exposure concerns raised by communities; that underestimates of exposure can lead regulatory agencies to conclude that unacceptable risks are, instead, acceptable, allowing pollutants to go unregulated; and that researchers, risk assessors, and policy makers need to better understand the issues that have affected exposure assessments and how appropriate use of exposure data can contribute to health-protective decisions.

**Methods:**

We describe current approaches used by regulatory agencies to estimate human exposures to environmental chemicals, including approaches to address limitations in exposure data. We then illustrate how some exposure assessments have been used to reach flawed conclusions about environmental chemicals and make recommendations for improvements.

**Results:**

Exposure data are important for communities, public health advocates, scientists, policy makers, and other groups to understand the extent of environmental exposures in diverse populations. We identify four areas where exposure assessments need to be improved due to systemic sources of error or uncertainty in exposure assessments and illustrate these areas with examples. These include: (1) an inability of regulatory agencies to keep pace with the increasing number of chemicals registered for use or assess their exposures, as well as complications added by use of ‘confidential business information’ which reduce available exposure data; (2) the failure to keep assessments up-to-date; (3) how inadequate assumptions about human behaviors and co-exposures contribute to underestimates of exposure; and (4) that insufficient models of toxicokinetics similarly affect exposure estimates.

**Conclusion:**

We identified key issues that impact capacity to conduct scientifically robust exposure assessments. These issues must be addressed with scientific or policy approaches to improve estimates of exposure and protect public health.

## Introduction

There is strong evidence that exposure to industrial chemicals, environmental pollution, agrochemicals, and chemicals leaching from everyday consumer products increases the risk of adverse health outcomes [1–5]. With this conclusion, there is growing concern that the methods used by regulatory agencies to conduct risk assessments are not sufficiently protective of public health [6–9]. Truly public health protective regulations would identify the exposures and exposure levels associated with harm and implement mitigation strategies to prevent such exposures before harmful effects occur. Associations between environmental chemical exposures and disease/dysfunction have been demonstrated in hundreds of environmental epidemiology studies, suggesting a failure of regulatory agencies to appropriately identify hazards [10], an inability to identify exposure levels that cause harm and increase risk, and/or systemic failures in risk management. These failures can be amplified by corporate efforts to hide research results and further lobby regulatory bodies to diminish their potential findings and regulations.

Characterizing and quantifying environmental exposures is critical for numerous elements of the policy process and for the broader protection of public health [11]. First, exposure assessments are important for understanding where interventions should be targeted. For example, the evaluation of lead in water, and in human blood samples, was essential for identifying sources and quantifying the extent of lead contamination during the Flint water crisis [12]. Exposure assessments are also important for determining the extent to which policy interventions may have achieved progress (e.g., in evaluations of the health risks remaining after the implementation of national air toxics emission standards for major industrial polluters such as coal-fired power plants [13]) or where further intervention is needed (e.g. in the evaluation of regrettable substitutes such as the replacement of bisphenol A (BPA) with other bisphenol analogues [14] or polybrominated diphenyl ether (PBDE)-based flame retardants with other toxic flame retardants [15]). Second, as noted above, evaluation of exposures is a critical component of risk assessments, which utilize exposure data to determine risks in the general population or specific subpopulations. Third, exposure data are often very important for communities, advocates, and other groups to understand the extent of a contamination; participating in the collection of exposure data can allow individuals and communities to be involved in studies that impact their health. For example, documentation of per- and polyfluoroalkyl substance (PFAS) exposures in communities has promoted additional evaluations of exposure sources (e.g., ground water, sludge, food) and demands by health advocates for stricter regulations and even bans of these chemicals in various states [16]. Finally, exposure data alone have sometimes been sufficient to trigger regulations of environmental pollutants. For example, in the European Union, implementation of a “Drinking Water Directive” (98/83/EC) requires pesticide concentrations in drinking water to remain below 0.1 μg/L for individual pesticides and 0.5 μg/L for the sum of all pesticides. The detection of atrazine in drinking water in a number of EU countries triggered restrictions or bans on its use [17].

Exposure assessments could therefore provide powerful tools by which affected communities, populations, and individuals can understand their quantified exposures and seek appropriate remedies, including protective government regulations. Health effects associated with exposures are often discovered years after exposures first occurred, and errors or uncertainties in exposure assessments can contribute to underestimates of exposure, detract from the validity of risk assessments, and put the public at risk. Thus, there is a need for exposure assessors to consider approaches that account for challenges in fully identifying and quantifying exposures and for policymakers to understand the limitations of exposure assessments [18].

In this review, we describe current approaches used to estimate human exposures to environmental pollutants. We then examine several weaknesses and problems with exposure assessments, especially as they relate to environmental chemicals regulated by the US Environmental Protection Agency (EPA). To illustrate how exposure assessments have reached flawed conclusions, we present a series of brief case studies. Finally, we conclude with some recommendations for how exposure assessments, and the use of exposure data, can be improved.

## Brief background on exposure assessments

Exposure assessment involves the identification and quantification of agents in the environment, allowing for the evaluation of the magnitude, duration, and frequency of exposure in a target population. As noted by the National Academy of Sciences panel on Exposure Science in the twenty-first Century [19], there are specific terms that are used to describe the various features of “exposure” (see Table 1).

**Table 1:** Key terms

**Key Terms**	**Explanation**
Environmental concentration	The amount of a chemical or environmental agent that is measured in an environmental medium at a specific place and time
Exposure pathways	The specific media through which exposures can occur (e.g., indoor and outdoor air, soil, dust, water, plants, animals, meat, dairy, fish, etc.)
Exposure routes	The ways that a chemical crosses an external barrier. Typically includes ingestion, inhalation and dermal exposure. Medical devices, regulated by the US FDA, can involve other internal exposures
Exposure scenarios	Provides information about chemical concentration, frequency of exposure, and duration of exposure as well as information about behaviors and characteristics associated with a specific life stage
External exposure	The amount of chemical present at an external barrier; sometimes referred to as the applied dose or administered dose
Internal exposure	The amount of chemical that crosses the external barrier, allowing it to reach (and, often, be measured in) internal tissues and fluids such as blood or urine. Sometimes referred to as dose, internal dose, or absorbed dose
Target site exposure	The amount of a chemical that reaches the tissue or organ where a biological action occurs

**Fig. 1:**
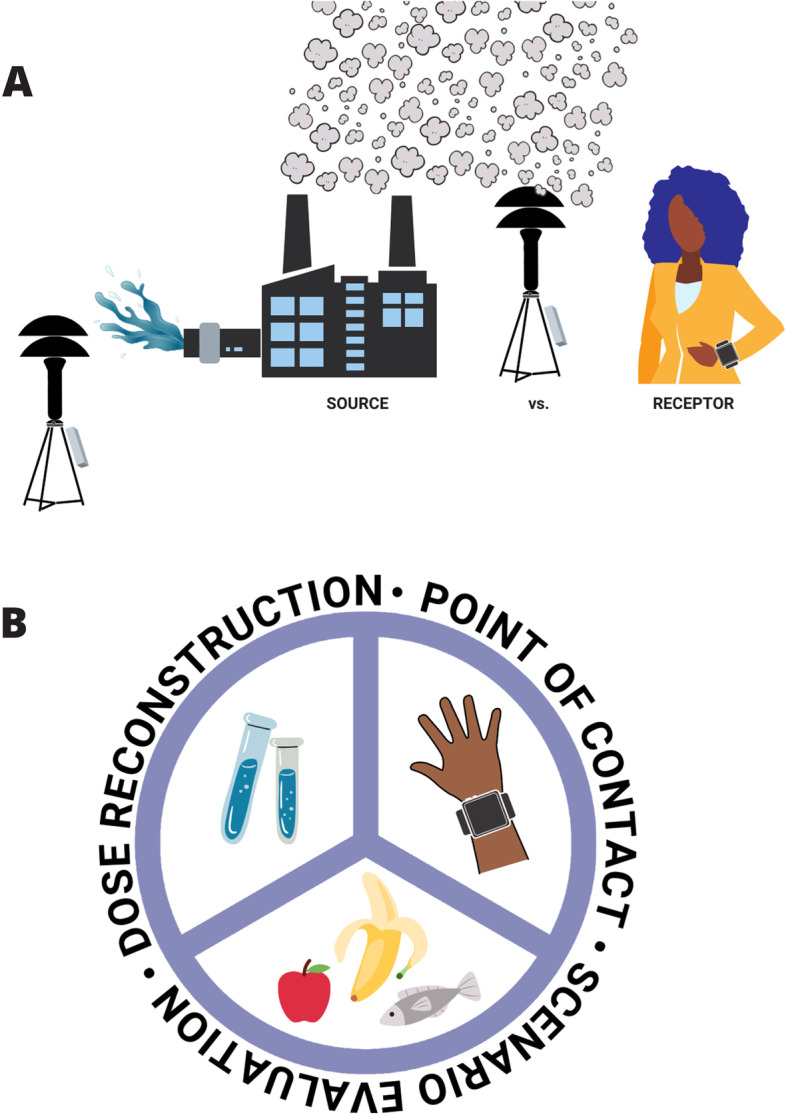
Typical approaches to exposure assessment. **A** Exposure assessments typically evaluate pollutants at the source, or at the receptor (e.g., in or near the bodies of people or animals that are potentially exposed). **B** There are three typical methods used to evaluate exposures of human populations: 1) Point of contact approaches utilize sensors placed near the individual to estimate exposures; 2) Scenario evaluation approaches measure pollutants within specific matrices (food, air, dust, water, etc.) and apply models of typical human interactions with those matrices to estimate daily intake; 3) Dose reconstruction approaches quantify pollutants in biological samples (urine, blood, etc.) and use toxicokinetic models to calculate daily intakes

Exposure assessments can evaluate and quantify environmental pollutants at (or near) the source where they are produced and/or released (Fig. 1A). For example, the EPA regularly provides public information about the emissions of sulfur dioxide (SO_2_), nitrogen oxides (NO_x_), carbon dioxide (CO_2_), and mercury (Hg) from fossil fuel-fired power plants [20]. These measures focus on the “source” of the environmental pollution. Source-focused exposure assessments often rely on data of how individuals interact with environmental media, as well as information about typical human behavior patterns, to estimate exposures in a target population. Source-focused exposure assessments are sometimes required by law (e.g., hazardous air pollutants in the Clean Air Act) and can be valuable in demonstrating the need for regulation or stricter pollution control. For example, the National Air Toxics Assessment (NATA) conducted every three years by the EPA uses air pollution emissions data from point and non-point sources around the country to provide estimates of air pollutant concentrations at the census tract level, which can be used to prioritize pollutants and types of emissions sources; an additional component of NATA integrates the ambient pollutant concentrations with human behavior patterns (i.e., empirical data used to evaluate activities in the exposed population) to estimate exposures [21].

There are several flaws in source-focused exposure assessments that can lead to underestimation of exposures. One major source of uncertainty in the current approaches used is that there are often insufficient measures of pollutants to extrapolate data across locations (e.g., air monitors can be placed in cities, but air pollution measures can be different from block to block depending on traffic patterns or even at different heights, accounting for differences in exposures between children and adults) [22]. Another limitation of source-focused exposure assessments is that these approaches are primarily focused on substances that are designated by the EPA as hazardous air pollutants (HAPs), but only 188 chemicals or chemical groups are currently listed HAPs; EPA has only proposed to add one chemical to the HAP list since the 1990 Clean Air Act amendments. This means that many chemicals that are not HAPs are released without monitoring, such as PFAS. A third contributor to underestimates of exposure in some source-focused assessments is that they often fail to account for aggregate exposures. In other words, they may not consider all sources of exposure (e.g., when calculating cancer risks of HAPs, the NATA does not account for exposures via indoor hazards, foods, etc.) [21]. For other kinds of exposures, such as those occurring from use of consumer products, there are also concerns that there is insufficient characterization of many typical (and atypical) human behavior patterns [23]. These flaws can lead to underestimates of variability in exposures, especially at the higher end of the exposure range.

In contrast to source-focused monitoring, three general approaches can be used to calculate exposures in (or nearer to) the bodies of individuals and/or human populations (sometimes referred to as “receptors”) (Fig. 1B) [24]. First, using the point of contact approach, measurements are made at the boundary of the individual and the environment (e.g., using personal monitoring approaches) and this information is combined with contact duration to calculate exposure [25]. This monitoring approach is often used to quantify exposures to noise, radiation, and some organic compounds in occupational settings. Wearable monitoring devices, including passive monitoring sensors, have become more common in observational studies to characterize a broader array of airborne environmental pollutants as well as exposures linked to use of personal care products, cigarettes, and pesticides [26]. Although there are many benefits to these kinds of passive monitoring approaches, they cannot assess all routes of exposure (e.g., they cannot evaluate all exposures via the diet) and they depend on the behavior of the user (e.g., wearers can choose to remove these sensors). Additionally, there is an expense trade-off in that passive monitoring approaches require sampling from many people to obtain accurate information as opposed to more central monitoring that can efficiently be used to estimate population-wide exposures.

The second approach, using scenario evaluations, combines data on the concentration of the chemical in an environmental medium with information on the frequency and duration of a typical interaction with that environmental medium, as well as additional information about the characteristics of the life stage when an exposure typically occurs. Formulas are used to estimate exposures based on typical rates of inhalation, ingestion, and/or dermal contact (Table 2). The EPA has created a series of “handbooks” to estimate how people typically interact with various environmental media and features related to where people spend their time (e.g., in settings such as home, work, or school) to estimate daily intake [24].

**Table 2:** Equations used in scenario-based calculations

Route	Calculation of Exposure
Inhalation	Exposure = concentration of a chemical in the air (mass per volume) x inhalation rate (volume breathed per unit time)
Ingestion	Exposure = concentration of chemical in food or other medium (mass of chemical per mass of medium or volume of medium) x ingestion rate (mass or volume of medium ingested per unit time)
Dermal	Exposure = mass of the medium (solid, liquid or gas) containing the chemical in contact with the skin x concentration of the chemical (mass of chemical per mass of medium) x skin surface in contact with the medium

Finally, using the dose reconstruction approach, exposures can be estimated based on measurements of biomarkers of exposure and physiologically based toxicokinetic (PBTK) models to calculate daily intakes of environmental pollutants. For example, PBTK studies of imidacloprid, an insecticide, were combined with urinary concentrations to model daily intake levels [27]. If biomonitoring data are available, the dose reconstruction approach is one way to aggregate exposures from all possible routes and sources; it offers the possibility to determine if there are unknown sources of exposure (e.g., if the amount of intake that is estimated based on the amount excreted exceeds the amount of intake from known sources). However, when there is incomplete or unavailable information (e.g., limitations in biomonitoring data or insufficient information around metabolism, kinetics, or distribution), PBTK models can have limited value. This is discussed in greater detail in *Problem 4,* below.

## The role for exposure assessment in scientific inquiry, risk assessment, and community-engaged research

### Exposure assessments in environmental health research

Exposure assessment is essential for several research fields in environmental health science. In *environmental epidemiology studies*, exposure data are used to evaluate associations between environmental agents and health- and disease-related endpoints. Correctly characterizing exposures is as important as the accurate characterization of outcomes. Historically, epidemiology studies have focused on single, or just a few, exposures [28]. However, increasing numbers of cohort studies are accounting for a more extensive number of environmental exposures with a holistic view of the exposome, e.g., the totality of exposures an individual experiences, from prior to birth throughout the lifespan [29, 30]. More modern approaches have also highlighted important challenges in the evaluation of exposures in human populations including the understanding and evaluation of how chemicals act in mixtures [11, 31–33]; an appreciation that people may be more sensitive to environmental perturbations during certain periods of life, but exposures may not be evaluated during these susceptible periods [34, 35]; differences in physiochemical properties that impact whether single or multiple biological samples are needed to properly characterize exposures (e.g., exposures to persistent chemicals can often be well characterized with single measurements whereas non-persistent chemicals may need multiple samples to fully characterize exposures); and also whether internal or excreted measurements are more appropriate [36].

Exposure assessments are also important to *toxicology* and other experimental fields where agents are administered in controlled settings. Some studies aim to test “human/environmentally relevant” doses in animals yet there are conflicting views of how to best replicate human exposures including challenges that arise from physiological differences between the animal species used in toxicity testing and humans [37]. In some studies, environmentally relevant doses are defined as doses administered to animals that are as similar as possible to measures of human intake [38] whereas others account for differences in toxicokinetic parameters between species to adjust the administered dose levels in rodents.

### Exposure assessments for risk assessment

Risk is informally described as the likelihood that harm will occur, and thus is often described as a measure that considers both hazard and exposure. Therefore, good estimates of exposure are essential for a risk assessment to be public health protective. Conversely, as described in more depth later in this manuscript, inadequate or error-prone means of evaluating exposures produce risk assessments that result in decisions that leave the public’s health at risk. Because exposure assessment data are used to determine whether the general population as well as specific subpopulations are at risk, it is essential that these assessments are as reliable and accurate as possible and avoid underestimation of exposure.

### Exposure assessments for communities

Civic science projects (sometimes referred to as “citizen science” projects) can involve exposure assessments conducted, at least in part, by community participants. Studies involving civic science exposure assessments span a number of environmental health topics, including the impacts of the Deepwater Horizon oil disaster on fishing communities [39], the quantification of heavy metals in water used by Native American tribes [40] and the consumption of fish by tribal populations in heavily polluted areas [41], and others. The development of relatively inexpensive sensors for environmental pollutants has made civic science projects even more feasible and allowed for communities to be active participants in the collection of exposure data; as described above, passive sampling monitors allow communities to evaluate pollution releases near manufacturing facilities [42–44].

There are also examples of exposure data being collected from isolated communities to promote environmental remediation and reduce exposures to harmful environmental agents. One example comes from the Bunker Hill Superfund site in northern Idaho, which was heavily contaminated by lead, zinc, and other chemicals released from a smelter site and a lead mine. Studies designed to characterize sources and levels of exposure documented high blood lead levels in the children in the 1970s (> 75% of children exceeded 40 μg/dl blood lead), high contamination of soil and wildlife, and high concentrations of lead in household dust [45–47]. Remediation efforts were taken and over a period of several decades the percentage of children with blood concentrations > 10 μg/dl decreased (from over 40% in the late 1980s to under 5% in the early 2000s) [48, 49]. The community affected by the Bunker Hill smelter and lead mine also contributed knowledge of factors that influenced the collection of community exposure data; for example, there was stigma and shame associated with lead exposures, which influenced the willingness of parents to have their children’s exposures evaluated [50].

Studies of the Bunker Hill Superfund site also revealed important ethical issues that have broader implications for exposure assessments conducted in heavily polluted communities [51]. First is the concern that public health actions taken based on exposure data are reactive, rather than proactive, and therefore illustrate a failure of risk management. Once a community has been found to have high exposures to known human hazards (like lead, dioxins, PCBs, etc.), then an essential time to prevent harm to the community has already passed, and damage has already been done. Thus, any public health actions taken within these communities are effectively damage control to prevent or reduce further exposure and harm.

When communities learn of documented high exposures to compounds with *unknown* or poorly characterized hazards, it often causes outrage and alarm [52, 53]. Compounding this alarm is the assertion from regulators that these exposures can be ignored because there are no *known* hazards associated with the contaminant, or because exposures are at a level that a government agency assumes to be safe (often based on incomplete or inadequate analyses of hazards). Ultimately, this response confuses ignorance of harm with the absence of harm and contributes to communities’ mistrust in regulatory agencies and scientists.

Communities that are designated Superfund sites are usually contaminated with multiple pollutants, and thus co-exposures occur. Furthermore, many communities that are challenged by environmental pollution are also affected by non-chemical stressors (e.g., increased disease prevalence, psychosocial stress from racial injustice, lack of access to green space). These stressors can exacerbate the effects of exposure and/or can directly contribute to increased exposures.

Communities can also be frustrated by the use of ‘action limits’ or ‘standards’ that are based on outdated science or economic concerns, or those that prioritize feasibility or practicality over health protection [51]. In the case of lead contamination, effects on neurodevelopment and IQ have been documented at exceptionally low blood lead concentrations, leading the US Centers for Disease Control and Prevention (CDC) to determine that there is no safe level of exposure [54]. Yet, remedial action objectives for Superfund communities like the Bunker Hill site in Idaho set goals of reducing children’s exposures so that at most 5% of the population will have blood lead levels above 10 μg/dl. Reaching this goal could still allow one in 20 children to experience exposures that would be considered high in the context of other (less exposed) communities and leave many other children with blood lead levels that will have adverse effects [55]. Lead is also an exception in terms of the extensive science available on its hazards; most environmental pollutants are much less well studied.

Finally, communities respond with frustration when exposures to environmental hazards are disproportionately experienced by populations based on social determinants of health (e.g., differences in socioeconomic status, education level, race and ethnicity, and other features that affect a community’s power and influence over decision-makers). This is especially true when community members feel that they were not responsible for the pollution but bear the burden of chemical exposures and their adverse impacts. In sum, there are some communities that are more heavily exposed to environmental pollutants, and these communities benefit from the use of the best available science to characterize exposures. These communities should also be involved in decision-making about how limited resources should be allocated to control or address hazardous exposures.

## Flawed exposure assessments put public health at risk

When exposure assessments are well conducted, they often include data that allow individuals or populations to act against exposures that are associated with excessive risk and allow for the promotion of exposure mitigation strategies that can be implemented at higher levels of the social ecological framework (e.g., interventions targeting whole communities, or reductions in exposure via pressure on corporations and/or bans and regulations). In this section, we evaluate several broad problems with exposure assessments and provide case studies that illustrate these points.

### Problem 1: With increasing numbers of chemicals released into the environment, many exposures are unknown

The Toxic Substances Control Act (TSCA) inventory of chemicals, overseen by the US EPA, includes more than 42,000 chemicals currently in use [56]. The vast majority of these chemicals have never been evaluated by regulatory agencies for exposure, hazard, and/or risk. With hundreds of new chemicals being introduced to the US market each year [56], and unknown volumes of release for many of these chemicals into the environment, neither exposure assessments nor risk assessments as currently conducted can keep up with this pace [57]. This creates constant and emerging needs for exposure assessments and data gaps that are unlikely to ever be filled under our current regulatory and policy structure.

For exposures that are evaluated using human biological samples, estimates suggest that we have the ability to measure and quantify fewer than 10% of all high-production volume chemicals in the US [58]. Furthermore, biomarkers of exposure in an organism (e.g., measurements of the chemical pollutant, metabolites of the chemical, or another fingerprint of exposure such as persistent changes in other small molecules such as pyruvate, glutamate, or amino acids that are distinct from the pollutant itself) are limited for most chemicals [59]. There are especially large data gaps for how biomarkers are influenced by dose, time, co-exposures, and non-chemical stressors.

There are several major issues that are introduced by the problem of the increasing universe of chemicals. One is the concern that chemicals are typically evaluated one at a time, rather than in environmentally-relevant mixtures [60], or by groups or classes (this is further discussed in the companion paper on chemical classes by Maffini et al. in this issue). There is evidence that chemicals can have additive effects within chemical mixtures; synergistic effects are rare but have been observed for a small number of pesticides, metal compounds, and some endocrine disrupting chemicals [61]. Most regulatory agencies also lack guidance documents that reflect the best available science for conducting cumulative risk assessments where the combined risks to human health or the environment are evaluated for multiple agents or stressors [62]. This hampers an agency’s ability to conduct robust cumulative risk assessments, which is concerning because single chemical exposure approaches are likely to underestimate risks by failing to account for simultaneous exposures to the variety of chemicals that people routinely face [63, 64].

Individual chemicals that are identified as hazardous can be replaced with other chemicals with unknown hazards; when those replacements are later found to be hazardous, they are described as regrettable replacements (or by the similar name, regrettable substitutions) [65, 66]. As chemicals are phased out of use, their replacements are often structurally similar analogues (e.g., bisphenol S [BPS] replacing BPA or the phthalate DINCH replacing diethylhexyl phthalate [DEHP]). Thus, as exposures to the first-generation chemical appear to be decreasing in a population, exposures to the second generation of chemicals – and there are often several replacements rather than a single one – may increase in the same population [67–69]. By the time that the hazards associated with the replacement are documented, exposures may be widespread. A relevant example comes from the study of polybrominated diphenyl ethers (PBDEs), a class of brominated flame retardant chemicals that were widely used in electronics, furniture, building materials, and children’s products [70]. By the early 2000s, the PBDE pentabromodiphenyl ether (Penta), which was widely used in polyurethane foam in furniture, was phased out of use voluntarily in European markets as evidence from rodents suggested it would have developmental and neurological effects on children [71] and it was widely detected in human breast milk [72]. Around that time, a US manufacturer introduced a replacement for Penta called Firemaster 550 [73], and an EPA news release suggested that Firemaster 550 was an environmentally-friendly replacement for Penta that would not bioaccumulate nor biomagnify [74]. Unfortunately, that prediction was not supported by the data [75]. While exposure assessments evaluating Penta and other PBDEs documented that human exposure levels have decreased in the last decade [76, 77], “novel” brominated compounds later determined to be chemical components from Firemaster 550 were detected globally in house dust and wildlife [78, 79]. Furthermore, in hazard assessment studies, Chemtura, the manufacturer of Firemaster 550, found that there were adverse developmental effects induced in rodents after exposures to the chemical mixture, but claimed there was “no risk” because the chemicals do not escape from treated foams (i.e., the report stated that their study “showed no detectable migration from the foam” [80]). The Chemtura study, as described by *The Chicago Tribune,* drew this conclusion using an inadequate attempt at an exposure assessment study. Filters soaked in saline were placed on cotton-covered blocks of foam made with Firemaster 550 and left there for 8 days. When the chemical constituents of Firemaster 550 were not transferred to the saline-soaked filters, Chemtura scientists claimed that human exposures also would not occur when using the foam. However, this is a scientifically inappropriate point-of-contact study because it is not representative of how exposures would occur in humans and does not replicate how the product is typically used; foams release chemicals into the air and dust as they are compressed through use and people are exposed to their flame retardant constituents via hand-to-mouth activity, dust ingestion, other dermal contact, and inhalation exposures [81]. This is also an example of a study conducted by the manufacturer of a product, where the results support the manufacturer’s position; these kinds of studies with inherent conflicts of interest are unfortunately common in environmental health sciences, including studies involving exposure assessments [82–84].

Another related, but separate issue, is the fact that the identities of thousands of chemicals remain unknown to the public because they are claimed as confidential business information and thus can be protected as trade secrets. An evaluation of 22 chemical inventories from 19 countries and regions revealed that more than 50,000 chemicals have been registered for use without disclosing their identities [56]; and in the US, the identities of more than 10,000 chemicals in the EPA’s TSCA inventory are considered confidential business information. This makes it difficult to predict and quantify exposures from consumer products, especially because there are inadequate disclosures of how chemicals are used throughout the marketplace and inadequate reporting of personal exposure data [85, 86], compounded by complex supply chains allowing chemical identities to be lost along the chain, e.g., manufacturers can use intermediates without full ingredient disclosure. Similarly, data needed for exposure assessments (e.g., the identity of all chemicals used in mixtures) can be considered proprietary or protected as trade secrets [87, 88].

The absence of analytical standards for many of the chemicals in commerce also makes exposure assessments nearly impossible, impeding scientific progress. For example, as early as the 1970s, researchers postulated that perfluoroalkyl substances were contaminating the bodies of humans and wildlife as well as ecosystems. Yet, this research did not progress until the early twenty-first century, when analytical standards for perfluorooctanoic acid became available [89]. Once available, analytical methods confirmed that exposures were widespread.

In sum, there are numerous problems that contribute to the “unknown unknowns” of exposure assessments. This includes the large number of chemicals currently on the market that are not well evaluated by regulatory agencies, including feasibility issues that limit the number of chemicals examined in biomonitoring studies; the failure to evaluate cumulative exposures, exposures to mixtures, or exposures across chemical classes; the replacement of one concerning chemical with other poorly studied substitutes; the use of ‘confidential business information’ designations to protect the identities of chemicals in consumer products; and the lack of analytical standards for many emerging pollutants. Collectively, these challenges mean that it is not possible to keep pace with new chemicals as they are introduced to the market [57].

### Problem 2: Exposure assessments become quickly out-of-date and are often not evidence-based

Exposure patterns and quantities change, yet exposure assessments are often static in time. This is especially true for new chemicals entering the market, where exposure patterns are not yet understood. For many chemicals, there is no requirement for companies to conduct post-marketing surveillance or to disclose how chemical usage changes over time; thus, as the quantity of a chemical being used changes, or that chemical is incorporated into new products, new exposure assessments are needed. An example of this issue comes from glyphosate, one of the most widely used herbicides to control weeds during crop production. Between 1971 and 2014, nearly 1.6 billion kilograms of glyphosate were applied in the US alone, and 66% of the total volume produced was applied between 2006 and 2016 (the last year evaluated) [90]. One explanation for the increased production and use of glyphosate is its role in the production of genetically modified crops; soy, corn, and other staple crops have been manufactured to be resistant to the herbicide, so that it can be widely applied to fields and weeds can be killed without harm to the crop [91]. Glyphosate and its major metabolite AMPA have been detected in historical urine samples collected from farmers starting in the late 1990s, documenting the presence of glyphosate prior to the widespread development of genetically modified crop technologies [92]. However, both the frequency of detection and the concentrations of both glyphosate and AMPA have increased in convenience samples collected since the 1990s. Despite this widespread use, few biomonitoring studies have evaluated human exposures to glyphosate [93]. Perry and colleagues suggest this is likely based on prior assumptions that glyphosate is non-toxic [92], which is being challenged by more recent toxicity tests and the International Agency for Research on Carcinogens (IARC)‘s classification of glyphosate as a probable human carcinogen [94, 95].

Even when it is clear that a chemical is being used in a specific product or function, it is often unclear how that product is actually used by broad groups of consumers or specific groups of targeted users. This is again highlighted by the example of glyphosate; as Perry and colleagues wrote, assumptions about the innocuous nature of the herbicide were based in part on use data suggesting that this chemical was applied only to crops pre-emergence (e.g., with typical application prior to planting, or long before crop harvest), without any risk of contributing to residues in food [92]. Yet, information provided in trade industry publications indicates that in addition to their use as weed-control herbicides, glyphosate-based herbicides were recommended for use as desiccants (on non-genetically modified crops) prior to harvest to accelerate natural drying of seeds and food crops (e.g., wheat and oats) [96]. Because the use of glyphosate-based herbicides as desiccants was not widely acknowledged until recently, it is unclear the degree to which this change in use pattern contributed to the increase in glyphosate production/use and subsequent exposure between the 1970s and today. However, this alternative use pattern likely contributes to the detection of glyphosate in harvested crops, and its later detection in food products such as cereals, which will contribute to dietary uptake in the general population [97, 98]. In addition to dietary exposures, the use of glyphosate-based herbicides in residential settings as lawn care products also contributes to widespread population exposures, even though such uses have been largely disregarded or ignored in exposure assessments.

Finally, there are also concerns that important features of the exposure assessments are not based on empirical evidence. For example, the EPA has published exposure factor handbooks that provide guidance on use and consumption patterns for many common exposures [24]. However, these guides also include estimates that are not necessarily based on empirical test data.

### Problem 3: Exposure assessments often rely on inadequate models and default assumptions, which can underestimate exposure

Exposure assessments often rely on assumptions for how people utilize products, or encounter media, that are outdated or do not consider contemporary knowledge of product use, or do not consider all (reasonable and anticipated) uses of a product. The example of thermal paper handling illustrates this point. In its exposure assessment for BPA, a chemical widely used in plastics, epoxy resins, and as a developer in thermal receipt papers, the European Food Safety Authority (EFSA) estimated that typical handling behavior for thermal paper for adolescents and adults was a single event per day, lasting for 10 seconds; high exposures were estimated at 4.6 handling events per day [99]. Furthermore, EFSA estimated that typical exposure involves touching of only three fingertips on one hand, whereas high exposure involves touching of three fingertips on both hands (six fingertips total). These estimates were challenged by the results of an observational study of individuals in a short-order cafeteria, where individuals order food and then are given a receipt while they wait for their food to be prepared. In this study, the average contact time was 11.6 minutes over a single handling event, with the majority of individuals having contact with fingers and the palm of the hand, quite unlike the assumptions followed by EFSA [100], even though the scenario of holding a receipt for an extended period while waiting for food to be prepared is likely a common experience. Equally important were the abnormal handling patterns observed in 2% of individuals, which included blotting lipstick, placing of the receipt in the mouth, using the receipt to remove food from the faces or hands, using the receipt to blot grease from food, or using the receipt to blot a wound. Although these actions are “unintended uses” in the context of regulation, they should be viewed as “reasonably foreseeable”, and considered for their potential contribution to the very high levels of exposure documented in human biomonitoring studies, usually in the top percentiles of exposures [101]. Furthermore, even though these actions occurred at low frequency (2% of individuals), because exposures are almost universal, large numbers of individuals are potentially displaying similar behaviors, leading to their high-level exposures.

Problems can also arise from inadequate quantifications of the measures of environmental chemicals in products or other media, as well as other factors that are relevant to internal exposure. Again, thermal paper highlights the challenge of extrapolating from human interactions with a product containing an environmental chemical, and quantifications of exposure (e.g., across the dermal barrier). Concentrations of BPA reported in peer-reviewed studies of thermal paper range from 0.211 mg/g to 26.3 mg/g, suggesting large variability (100-fold) across products [100]. Similarly, transfer rates used to estimate the migration of BPA from thermal paper to skin have ranged from 1072 to 21,522 ng/sec, and absorption factors for BPA in skin range from 2.3 to 27% [102, 103]. All of these values suggest large amounts of uncertainty in estimations of daily exposures from the handling of thermal papers (Table 3).

**Table 3:** Estimates of BPA in thermal paper from published studies versus values used in exposure assessments by the European Food Safety Authority

	Liao & Kannan [104]	Geens et al. [105]	Zalko et al. [103]	Demierre et al. [106]	Hormann et al.	Biedermann et al. [102]	Bernier & Vandenberg [100]	EFSA [99]
Concentration in thermal paper (μg/g)	211–8880	21,000			19,600–26,300	13,000		
Transfer coefficient (ng/s)					1072–1838	21,522		
Absorption fraction			46–65%	2.3–8.6%		27%		10%
Length of typical handling (sec)							690 ± 16	10 (typical) 46 (high)
Area of typical handling event (cm^2^)							14.15	2 (typical) 9.0 (high)
% individuals with abnormal handling							2%	Dismissed as irrelevant
Estimates of daily intake from thermal paper handling, adults only (μg/kg/day)	0.0175–1.3	0.0064					0.051–1484	0.059 (average) 0.542 (high)

Various parameters relevant to human exposures to BPA from the handling of thermal papers have been measured in different model systems and across studies (shown in the first seven columns). EFSA’s exposure assessment model is shown in gray. EFSA dismisses abnormal handling (e.g., children chewing on thermal paper) as irrelevant. Other parameters selected by EFSA (e.g., absorption fraction, length of typical handling time, area of typical handling event) differ from what has been observed in human populations; EFSA also does not report which concentrations of BPA measured in thermal paper were used in their estimates

Furthermore, exposure pathways can be missed, leading to underestimates of exposure or an inability to identify highly exposed populations. This issue is compounded when there is poor characterization of a chemical in the medium of concern. An example comes from the evaluation of exposure routes to phthalates. Prior to 2012, it was assumed that dermal uptake of these chemicals required contact with the skin. Yet, a remarkable study using human volunteers who spent 6-hour periods in a chamber with elevated air concentrations of two phthalates demonstrated that simply sitting in a room with phthalates in the air could increase urinary concentrations, even when clean air was provided for breathing [107]. Based on this novel exposure assessment approach, it was estimated that up to 25% of daily exposures could be due to inhalation and dermal uptake from the air.

Although EPA assesses multiple pathways and routes of exposure, the agency often fails to aggregate the exposures it finds in risk assessments. EPA instead creates exposure assessments based on sources and pathways that are in the jurisdiction of a particular environmental statute (e.g., the Clean Air Act), the pathway that is the greatest contributor to exposure, or the source estimated to affect the most number of individuals [108]. Regulatory agencies like the US FDA and EPA also often fail to aggregate exposure pathways that are regulated by other agencies (e.g., cosmetics are often ignored in exposure assessments conducted by the EPA whereas consumer products are often ignored by the FDA) [109].

### Problem 4: Toxicokinetic knowledge is incomplete, and PBTK models can be overly complicated, obscuring underlying data inadequacies

Rodents and other experimental animals are often used to understand toxicokinetic patterns following chemical administration [110]. These studies are useful to understand whether chemicals bioaccumulate, to identify the typical routes of excretion, and to assist researchers in identifying the areas of greatest uncertainty. Yet, there are important differences in toxicokinetics between species, and thus data from humans are often needed to construct accurate and relevant models [111].

Unfortunately, PBTK modeling can be used to inappropriately downplay or dismiss concerns about exposures, e.g., by suggesting that exposures of internal organs are at levels “insufficient” for harm to occur, even when epidemiology or toxicology evidence suggests otherwise [112]. For example, a PBTK model that was created for chloroprene, a volatile chemical used in the production of synthetic rubbers, used in vitro data to conclude that a reactive, oxidative metabolite is fully consumed in the tissues where metabolism occurs (e.g., liver and lung) [113]; yet, chloroprene administered to rodents induces tumors in the mammary gland (and other sites), suggesting that the toxic metabolite circulates through the bloodstream to these distal locations [114]. An external review of the chloroprene PBTK model concluded that it relied on unsupported assumptions; although one reviewer wrote in his comments that the PBTK model for chloroprene is “potentially useful”, he also noted that “the objective of [PBTK] models is to fit the data, ignoring and often hiding basic scientific principles and including ‘fudge’ factors…” and “If [PBTK] models have to be scientifically valid, we will have no useful [PBTK] models” [115]. Thus, even though PBTK modeling presents an opportunity to understand how and why some organs (or some life stages) might be more vulnerable than others, it should not be used to dismiss studies showing associations between exposures and health effects.

In humans, PBTK data are often lacking for many environmental pollutants; when available, models are typically built from data collected from young, healthy (often male) adults [116, 117]. This can leave data gaps in our understanding of how variability in physiological characteristics affects toxicokinetic parameters. PBTK models of exposures in the developing fetus are particularly inadequate. Thus, when measured *exposures* in individuals from other groups (e.g., children, pregnant women, elderly) diverge from the general population, it is often unclear if this is due to physiologically-based alterations in toxicokinetics, altered intake, or some other factor. An illustrative example of this situation comes from comparisons of biomonitoring data collected from pregnant women, compared to similar aged non-pregnant women [118]. Such comparisons have revealed higher exposures to DMTP (an organophosphate pesticide metabolite) and perchlorate in pregnant women compared to non-pregnant adults; in contrast, higher exposures to PFOS and BPA were observed in non-pregnant women compared to pregnant women. Are these differences between pregnant and non-pregnant women due to differences in external exposures, differences in uptake (e.g., due to altered permeability of membranes in the skin, gut, or respiratory tract), or alterations to other aspects of toxicokinetics (e.g., metabolism or excretion)? Appropriate PBTK data can help to address these questions, if such data exist, but PBTK data are unlikely to be collected in pregnant humans.

For many chemicals, even basic information on absorption, distribution, metabolism, and excretion (ADME) parameters is absent. This can cause problems when deciding which biological matrix is the correct one for biomonitoring studies (e.g., urine versus blood/serum/plasma) or determining whether the parent compound or its metabolites (one or several) should be measured. This issue is illustrated by the example of atrazine, an herbicide that is used to control broadleaf plants and is widely sprayed in the US on corn crops [119]. Historically, atrazine has been used heavily across the world, with reports in the early 1990s suggesting a worldwide application of 70,000–90,000 tons per year [120]. Even after bans in the EU, it remains one of the most commonly used herbicides in the US, China, and Australia [17]. Atrazine received significant attention because of its ability to alter sexual dimorphism in amphibians and affect male gonad health across vertebrate classes [121]. In spite of its very heavy use (in the US, it is the second most used pesticide after glyphosate), numerous biomonitoring studies (including several NHANES cohorts) have concluded that exposures to the general population are low, with relatively low detection rates between individuals. For example, in one study, researchers at the CDC found detectable levels of the atrazine metabolite, atrazine mercapturate, in the urine of fewer than 5% of the population [122]. In other studies, this metabolite was not detected in any individuals above the limits of detection. The conclusion that humans are not exposed to this pesticide is at striking odds with the use data. To address this disconnect, Barr and colleagues later determined that measurements of only a small number, instead of all of the known metabolites of atrazine, were leading to significant underestimates of exposure [123].

A similar problem was documented when evaluating metabolites of di-(2-ethylhexyl) phthalate (DEHP) in urine samples. It had been assumed that mono-(2-ethylhexyl) phthalate (MEHP) was the major metabolite of DEHP. Yet, early biomonitoring studies found lower levels of MEHP in urine samples than the measured concentrations of metabolites of other phthalates that were used in lower volumes than DEHP [124]. This led exposure scientists to identify two other metabolites of DEHP, mono-(2-ethyl-5-oxohexyl) phthalate (MEOHP) and mono (2-ethyl-5-hydroxyhexyl) phthalate (MEHHP), which were found at 10-times higher concentrations than MEHP in urine [125]. These results indicated that dose reconstruction studies relying on MEHP concentrations alone would lead to significant underestimates of exposure for DEHP. Such underestimates of exposure due to the use of only a subset of metabolites are no longer isolated cases. For example, assessments of paraben exposures often fail to measure major metabolites that can account for > 75% of the parent compound intake. The failure to include these metabolites in biomonitoring programs is certain to affect the accuracy of exposure assessments, but may also introduce bias in epidemiology studies that associate paraben exposures to human health outcomes, considering individual differences in rates of transformation of these compounds [126].

## Using policy to prevent exposures

Ultimately, exposure assessments can be used to help stakeholders to address concerning exposures – either because the exposures themselves are high, or because the risk assessment that uses the exposure data indicates that exposures should be mitigated. In some instances, as described in more detail below, the law requires that exposure assessments be used as a basis for regulating toxic chemicals and pollution. For example, Section 112(f)(2) of the Clean Air Act requires the EPA to prevent all “unacceptable” hazardous air pollutant risks to the most-exposed individuals and to assure “an ample margin of safety to protect public health”.

Originally enacted in 1976, TSCA is one of the most influential laws that governs chemicals in commerce, and it gives the EPA authority to regulate the manufacture and sale of many chemicals to protect the public from those that pose unreasonable risks, though there were numerous statutory language obstacles that did not allow EPA to fully use the science to prevent harmful exposures. In 2016, a revised TSCA created a framework that requires the EPA to evaluate safety for new chemicals, and a plan to prioritize and evaluate risks for chemicals already in commerce [127]. Numerous limitations have been identified in the implementation of the revised TSCA including flawed approaches for systematically evaluating evidence (including data relevant to both hazards and exposures) [128], insufficient resources to tackle the prioritization, evaluation, and assessment of the ~ 42,000 chemicals regulated by the EPA that were already in commerce at the time 2016 TSCA was passed [129], and preemption of state-level regulations [130]. While there are important limitations to the law, the 2016 TSCA offers some improvements over the prior TSCA law; for example, it explicitly directs the EPA to protect potentially exposed or susceptible subpopulations, defined as “a group of individuals within the general population… who, due to either greater susceptibility or greater exposure, may be at greater risk than the general population” (emphasis added [127]).

Beyond the role of laws and regulation, international treaties and protocols have been used to restrict or ban the use of hazardous substances on a global scale. For example, the Stockholm Convention, originally signed in 2001 but yet to be ratified by the United States [131], eliminated the production and use of persistent organic pollutants such as chlordane, dieldrin, polychlorinated biphenyls, and perfluorooctanoic acid, and restricted the use of others such as DDT and perfluorooctane sulfonic acid. Human exposures to many of these chemicals were well documented, both in environmental media and in biomonitoring samples [132]. Efforts taken by numerous nations to reduce the release of persistent organic pollutants, in response to the Stockholm Convention, led to decreased concentrations of many of these chemicals in both the environment and in human bodies [133, 134], indicating the effectiveness of these global approaches to control pollution.

Other approaches to reduce exposures have resulted from corporate reforms as a result of social movements, driven by environmental health and justice advocacy groups, to push for safer products [135]. In 2013, Walmart began requiring suppliers to disclose, and eventually commit to phasing out, 10 targeted chemicals from consumer products; the specific chemicals were not named by the company at that time. In 2015, Macy’s department store announced that it would phase out the sale of furniture products containing flame retardant chemicals. In 2020, Target promised to phase out bisphenols from the thermal receipt paper it uses. Each of these examples illustrates how consumer demands impact sale and use patterns in consumer goods. However, corporate reforms are susceptible to regrettable substitutions and thus require comprehensive approaches to evaluate the replacements that are proposed for use; for example, after PBDEs were banned and then replaced by other hazardous flame retardants, California responded in 2020 by restricting the use of flame retardants in specific consumer products including mattresses and upholstered furniture. Corporate reforms are also extremely uncommon for broader issues of environmental pollution (e.g., air pollution), and it can be challenging to determine if corporations comply with their own policies. Even well-intentioned companies lack the full knowledge of chemicals and contaminants used or produced along the supply chain.

## Recommendations to improve exposure assessment and its use in hazard and risk assessment

### Addressing scientific uncertainties in exposure science

From the scientific perspective, there are numerous exposure assessment data gaps that need to be filled. There are thousands of chemicals that should be measured in environmental media and in human tissues/fluids, but transparent and science-based methods are needed to prioritize the overwhelming number of chemicals to work within fiscal constraints for this work. Additional research is also needed to better understand the features of chemicals that influence ADME parameters, including how physiological changes and life stages can impact aspects of exposure and whether the parent compounds versus the metabolites can bioaccumulate or biomagnify in the food chain. We also need to create mechanisms to improve disclosures of chemical uses and require new exposure assessments when uses of a chemical change.

There is an urgent need to identify and protect the most highly exposed individuals and communities in the population for a larger number of chemicals; however, current exposure assessment data are insufficient to even determine the characteristics of those highly exposed individuals and communities. For many pollutants, the most exposed populations are low-wealth communities and communities of color, but for others, higher socioeconomic status has been associated with increased exposures [136]. Thus far none of the risk evaluations under 2016 TSCA have assessed differences in exposure along the parameters of race/ethnicity or income.

Disparities in individual and community exposures to environmental pollutants exposures cannot be divorced from extrinsic (e.g. psychosocial stressors such as racism and poverty) or biological susceptibilities (e.g. age or pre-existing conditions) that also contribute to health disparities and risks from environmental hazards; this is commonly known as the “triple jeopardy hypothesis” [137–139]. While intrinsic susceptibilities such as age and pre-existing disease are better characterized, extrinsic susceptibilities are less so. Some of these susceptibilities include violence (victim of, or witness to) [140]; issues with the built environment (e.g., poor housing quality, current land use management driven by historic redlining) [141]; socioeconomic status; educational attainment; exposure to segregation and racism; and poverty. Thus, to fulfill TSCA’s mandate to consider populations that are at greater risk from chemical exposure due to increased susceptibility (including to psychosocial factors such as violence, systemic racism, and poverty*)*, it is essential for more targeted analyses to be conducted in these vulnerable populations. It is also essential for EPA to quantitatively incorporate these factors into its risk assessment approaches, which can be done through the use of additional adjustment factors to account for uncertainty and within-person variability relevant to exposures. Further discussion around these adjustment factors is in the companion paper on human variability by Varshavsky et al. in this issue, but should at a minimum account for:

- Extrinsic factors affecting exposure. The “socio-exposome” approach considers features that can contribute to differences in exposures between subgroups or individuals, or prevent inequalities in exposures from being addressed [142] including violence, urban design problems, other issues with the built environment, segregation and racism, lack of green space, and poverty, among others.
- Mixture effects. Most exposure assessments consider single chemicals, even though the reality of exposures is a mixture. For those that do consider multiple chemicals, some components of the mixture will likely be unknown or excluded. Guidance has been published on how to account for exposure to mixtures in risk assessment [143, 144]. The National Academy of Sciences has also recommended application of dose addition methods to mixtures of chemicals that share “common adverse outcomes” [63], but these methods are infrequently applied. The total number of mixtures that can be experienced by individuals is close to infinite; thus, even an additive exposure assessment approach is insufficient to protect public health. For this reason, an additional adjustment factor should be included to account for the possibility that exposures to unknown chemicals in the mixture could alter metabolism of the chemical in question or could act additively with known chemicals, unless there are data to the contrary [64].
- The absence of data for exposures in certain age groups or across the life course. There are numerous examples of chemicals where exposures have been measured only in adults, and then assumptions are used to calculate likely exposures in infants and children. These assumptions are later shown to be insufficient to model the behaviors and other factors that influence exposures in infants and children (e.g. [145, 146],). Some state regulators such as California EPA (Cal EPA) have developed child-specific risk values for specific chemicals (e.g., atrazine, lead, nickel, manganese, heptachlor) that address routes of exposure (as well as other differences in susceptibility) that are unique to children compared to adults. At a minimum, EPA should start with Cal EPA’s age adjustment values and intraspecies uncertainty factors for incorporating age/early life susceptibility.
- Differences in exposures across physiological stages. In addition to an adjustment factor for the likelihood that *hazards* are different across different life stages, there should be adjustment factors that account for the fact that exposures can also differ significantly due to physiological status. As discussed already, there are chemical exposure differences between pregnant and non-pregnant adults that affect intake, internal exposure, and/or ADME parameters [147].
- Uncertainties relevant to poorly studied exposure routes/sources. The example of BPA uptake from thermal paper shows the considerable number of uncertainties relevant to parameters including transfer rates and absorption factors for dermal exposures, as well as the possibility of atypical exposure routes and sources. Ideally there should be studies and survey data regarding how individuals use and interact with various products in various exposure settings (like consumer interactions with thermal paper receipts, or workers’ use of protective gloves). These studies should be updated regularly because behaviors change over time. In the absence of such studies to inform exposure assessments, adjustment factors should account for uncertainties and the possibility of atypical exposures.

Furthermore, for some chemicals, regulatory agencies use probabilistic approaches, combined with the concentrations measured in an environmental matrix with data on human use, intake, ingestion, or inhalation of that matrix to use scenario approaches to calculate an exposure “threshold of concern” [148] for risk management consideration. This threshold of concern may be set at the 90th, 95th, 99th, or the 99.9th percentile of exposure (or intake). (These thresholds of concern are distinct from health effects as there is no population level threshold for health effects [149, 150]). Such probabilistic approaches often consider a single route of exposure, rather than an aggregate (or even a cumulative) exposure. They also do not typically consider exposures that occur due to “atypical” uses of consumer products by people that believe the product must be ‘safe’ (e.g., the use of thermal paper to blot grease from food). In the US, regulatory actions that protect individuals at the 90th or 95th percentile of exposure leave millions (or tens of millions) of Americans at risk, especially because there is widespread or even universal exposure to many pollutants. Because of ubiquitous exposures to many pesticides, for example, even the EPA’s approach which sets a regulatory threshold for action corresponding to the 99.9th percentile of intake [151] leaves tens of thousands exposed to potentially concerning levels. As noted in the example of glyphosate, the situation is made worse if the data used to calculate this intake level are underestimates due to changes in use patterns or environmental concentrations. As discussed more below, these may be cases where usage data should be sufficient to trigger regulatory efforts.

### Resourcing and funding mechanisms

In addition to updating current exposure methods, there is also an urgent need to develop sustainable resourcing and funding mechanisms for communities and environmental justice organizations to lead the work on environmental hazards in their own backyards; community members are the experts about the community’s experiences, and exposure assessments would be improved by incorporating their knowledge [152]. Community members should be empowered with data. For example, there is evidence that providing communities near a Superfund site with information about their own exposures allows their members to be involved in decisions about exposure mitigation efforts; in a Superfund community in Louisiana, USA, those community members with the greatest level of knowledge of the hazards present in their environment were the most likely to adopt behaviors that reduced their own exposures [153]. This can be accomplished by increasing the funding opportunities accessible to communities as well as removing obstacles to accessing such funding (e.g., through one-on-one grant counseling or assistance) and considering ways that community gathered data might be incorporated into ongoing assessments. Importantly, providing community members with information that they can use to change their own behaviors should not be seen as sufficient public health actions, because it should not be individuals’ responsibility to avoid hazards introduced by polluting industries.

Additionally, any funding opportunities around environmental justice for academic institutions or local governments should have an explicit requirement and funding for active community leadership and be driven by community questions, rather than the lower bar of community involvement. We must include diverse cultural perspectives in studies focused on exposures, which can only be achieved by valuing the leadership and knowledge within impacted communities. We also must build a more complete, “whole fabric” understanding of health effects of environmental exposures to chemicals and put special focus on understanding overlapping threats. This capacity building can begin to reverse systemic racial discrimination, close racial disparities in exposures, and decrease harms from contact with hazardous products on the market and their manufacture and disposal.

Agencies such as EPA must actively seek technical guidance from communities when developing improved mapping or screening tools to build a comprehensive understanding of the cumulative and disproportionate impacts of chemicals and invest in community based participatory research that is responsive to community needs and can inform EPA science and policies. In addition, they must accelerate environmental education programs with input from community experts.

Similarly, funding mechanisms are needed to support the development and advancement of exposure assessment methodologies. Such efforts should include the validation of exposure assessments that have already been conducted. Investments in these approaches are essential to ensure the use of modern approaches in both hypothesis-driven research and public health protective studies that contribute to risk assessments.

## When enough is enough

There are numerous examples (including atrazine, discussed above) where chemical production volumes are so high (i.e., they are produced or imported into the US in quantities of 500 tons per year or greater) that human exposures should be expected, at least in some groups or populations [154, 155] along the lifecycle of the chemical. In these cases, it would be prudent to revise regulations that formalize the use of chemical production volumes to trigger additional scrutiny and potential interventions; when manufacturers produce chemicals above a certain volume, consequences should then occur to quantify exposures, as well as a mandate to capture releases to the environment. However, in these cases where production volumes are high, calls for additional biomonitoring to verify that exposures are occurring can be seen as an excuse to delay action. We should not wait for more data to confirm what is almost certain: when chemicals are produced in high volumes, human (or environmental) exposures are very likely occurring. The constant call for biomonitoring data means that the protection of public health is perpetually delayed.

It should also be sufficient to show that a chemical (or its metabolite) is detected in human urine to acknowledge the reality of human exposures; thus, we should assume universal system/organ exposure based on measurements in urine/blood. It is not necessary to show that a chemical is present in a target organ (e.g., the breast) to conclude that exposure to that target organ has occurred.

It is also fundamental to acknowledge the needs of communities, especially those that are downstream of sites where pollutants are created and/or released. There are unfortunately too many examples where inadequacies in exposure data and methods underestimate exposures, and these data have been used to downplay and ultimately disregard concerns raised by communities experiencing these exposures firsthand. These communities are often told that the health effects observed in the community have not been *definitively* linked to the chemical contaminants (which may fail to acknowledge the role that data from communities can play in establishing such associations); they are told that mitigation is not possible due to cost or feasibility issues, or because there are no regulations that specifically require clean-up; they are told that addressing polluters could put the economic stability of their own community at risk; and they are told that their perceptions of environmental racism are misplaced [156–159]. Furthermore, communities often have interactions with regulatory agencies that leave their members feeling unheard, unseen, and disrespected [160]. The scientific needs of communities cannot be met if issues related to environmental justice, equity, and transparency are not addressed [161] and economic feasibility should not be an excuse to fail to apply the best available science and approaches to reduce total contaminant risks to levels that are acceptable to regulatory agencies (e.g., the EPA’s accepted lifetime *de minimis* risk of one in a million) and the community.

## Conclusions

Some of the biggest problems with exposure assessments, and how exposure data are used, will require changes at the highest socioecological levels of the public health framework including risk assessment and risk management. Changes are needed to address exposures to chemicals from products, exposures to pollutants that are broadly experienced by communities, and the methods by which exposures are evaluated by regulatory agencies. Additionally, while we have focused heavily on the impact of environmental chemical exposures on humans, the broader ecosystem is also impacted by chemical exposures [19] and detection of environmental pollutants should trigger remediation efforts as well as health-protective efforts.

In this review, we have highlighted three fundamental problems that influence our ability to develop and produce accurate and scientifically appropriate exposure assessments including the increasing number of chemicals being registered for use, both in the US and globally, which is outpacing the ability of regulatory agencies to conduct both exposure and risk assessments; how changes in use patterns can lead to underestimates of human exposure; and how exposure data can be concealed from the public if an industry claims them as confidential business information. All three of these issues need to be addressed with better oversight of the industries responsible for chemical production and release into the environment (including data sharing requirements). We also described how the use of inadequate models of product use (and/or models quantifying interactions with environmental media), as well as flawed PBTK models, can contribute to underestimates of exposure. These issues can be addressed via a reliance on the best available science. However, we continue to reiterate that calls for “more science” are often made with the intention of delaying regulatory action rather than improving scientific knowledge. Such delays must be avoided so that all communities, but especially those that are most vulnerable, can be protected from toxic chemicals and pollutants.

## Data Availability

Data sharing is not applicable to this article as no datasets were generated or analyzed during the current study.
